# The Utilization of Serum Folate and Homocysteine Tests and the Prevalence of Folate Deficiency in Reproductive-Age Korean Women during the COVID-19 Pandemic

**DOI:** 10.3390/nu15143236

**Published:** 2023-07-21

**Authors:** Rihwa Choi, Wonseo Park, Gayoung Chun, Sang Gon Lee, Eun Hee Lee

**Affiliations:** 1 Department of Laboratory Medicine, Green Cross Laboratories, Yongin 16924, Republic of Korea; pirate0720@naver.com; 2 Department of Laboratory Medicine and Genetics, Samsung Medical Center, Sungkyunkwan University School of Medicine, Seoul 06351, Republic of Korea; 3 Infectious Disease Research Center, Green Cross Laboratories, Yongin 16924, Republic of Korea; 4 Green Cross Laboratories, Yongin 16924, Republic of Korea

**Keywords:** folic acid, folate, homocysteine, reproductive age, Korea

## Abstract

We investigated the prevalence of folate deficiency and associated factors in a large population of Korean women of reproductive age during the COVID-19 pandemic. We utilized different cut-offs and evaluated age, year of testing, geographical region, and the utilization of serum homocysteine levels. Out of the 27,758 women evaluated, the overall prevalence of folate deficiency was 12.5% (<4 ng/mL, metabolic indicator) and 5.4% (<3 ng/mL, hematologic indicator). Homocysteine testing was observed in 8.4% of women, with 2.7% having elevated homocysteine levels (>15.4 µmol/L). According to our multiple logistic regression analysis, younger women, particularly those aged 20 to 24 years, tested in 2020, and from Jeolla province, Gyeongsang province, and Jeju Island, were identified as being more prone to folate deficiency. Receiver operating characteristic curve analysis demonstrated that a cut-off of >8.4 µmol/L provided the most accurate definition of folate deficiency with serum folate levels <4 ng/mL, while a cut-off of >8.8 µmol/L best defined folate deficiency with serum folate levels <3 ng/mL, with both cut-offs being lower than 15.4 µmol/L. Our study emphasizes the prevalence of folate deficiency, associated factors, and the role of homocysteine in planning nutritional support programs in Korea.

## 1. Introduction

Folate deficiency is a major global concern under the umbrella of maternal and child health in association with related health outcomes, such as neural tube defects [1]. Globally, the average prevalence of neural tube defects, including spina bifida and anencephaly, is about 20 cases per 10,000 births [1,2,3]. A recent study suggested that there is an urgent need for universal food fortification with folic acid to prevent birth defects, save lives, and promote health equity [1]. Maternal nutritional deficiency and supplementation status may have been impacted by the coronavirus disease 2019 (COVID-19) pandemic; however, regard-less of whether or not more women and their newborns were at risk of complications during the pandemic period, the nutritional effects and health outcomes of folate deficiency are of growing interest [4,5].

Folate deficiency can be assessed by biochemical analysis, focusing on serum and erythrocyte folate levels and other analytes in folate metabolism such as homocysteine and methylmalonic acid [6,7,8]. Folate, a water-soluble B vitamin (vitamin B9), is vital for multiple cellular processes, including DNA synthesis and repair, and acts as a cofactor for enzymes such as methionine synthase and L-methylmalonyl-coenzyme A mutase in the shared metabolic pathway with vitamin B12, facilitating the conversion of methylmalonic acid to methylmalonyl-CoA and Succinyl-CoA [6,9,10]. Homocysteine, a non-protein amino acid, is generated during methionine metabolism [6,8,11]. In cases of folate deficiency or vitamin B deficiency, the accumulation of homocysteine leads to elevated serum homocysteine concentration [6,8,11]. Furthermore, it has been suggested that there is an association between elevated serum homocysteine concentration and cardiovascular disorders, and extensive research has been conducted on the role of vitamin B supplementation in preventing atherosclerosis and other cardiovascular diseases [12,13,14,15].

Serum folate assays may not accurately indicate actual folate deficiency due to the impact of recent food intake, whereas homocysteine levels are utilized as a metabolic indicator for assessing folate deficiency [6,8,11]. However, the optimal cut-off for homocysteine levels to define folate deficiency is still under investigation, along with the standardization of analytical methods for quantifying serum folate and homocysteine [16,17,18,19,20].

Information regarding the global folate status in women of reproductive age is available through the World Health Organization (WHO) Micronutrients Database [21]. This database provides data on folate deficiency using serum and erythrocyte folate and homocysteine results [11]. However, the database has certain limitations, including a lack of information on the analytical methods employed for determining folate deficiency, limited standardization of the analytical assay, and variations in cut-offs for defining deficiency [18,21,22]. Additionally, data specific to Korean women of reproductive age are not accessible through the WHO Micronutrients Database.

Limited data are available regarding the prevalence of folate deficiency in a large number of Korean women of reproductive age. The recommended nutrient intake (RNI) levels for folate vary among nutritional guidelines in diverse ethnic populations [23,24,25,26,27]. For example, the recommended nutrient intake (RNI) for non-pregnant women in the USA is 400 µg/dietary folate equivalent (DFE) per day, which is the same level suggested in Korea, whereas it is 200 µg/day in the UK [23,24,25,26,27]. In Korea, the Ministry of Health and Welfare of South Korea and the Korean Nutrition Society periodically publish dietary reference intakes (KDRIs) that include current folate intake recommendations and information on food products, based on data from the Korea National Health and Nutrition Examination Survey (KNHANES) [23,28,29]. The KNHANES phase VII (2016–2018) included the measurement of serum folate, and information on the dietary intake of folate for Koreans based on KNHANES data was released to the public in 2019 [23,28,29]. According to the KNHANES phase VII (2016–2018) data, the overall intake of dietary folate in the Korean population was reported to be a mean of 295.5 μg DFE/day (standard error, SE 2.4) [23,28,29]. Based on KNHANES phase VII (2016–2018) data, the mean folate intake among Korean women of reproductive age was 239.2 μg DFE/day (SE 7.6) for those aged 19–29 years and 290.4 μg DFE/day (SE 4.3) for those aged 30–49 years [23]. In the USA, the mean (SE) total usual folate intake among non-pregnant, non-lactating women aged 20 to 44 years was 493 μg DFE/day (SE 7.24) in the group that consumed foods alone, and 671 μg DFE/day (SE 11.7) in the group that consumed both foods and supplements, based on data from NHANES 2007–2018 [30]. In the UK, the National Diet and Nutrition Survey reported that the median dietary folate intake in women of childbearing age, defined as aged 16 to 49 years, was 192 μg/day in survey years 9–11 (2016–2018) [31]. Based on KNHANES 2017 data, soybeans, eggs, spinach, white rice, and kimchi were identified as major contributors to folic acid intake in the Korean population [28,29]. Folate deficiency is known to result from insufficient dietary intake, intestinal malabsorption, increased physiological requirements, and the use of antifolate drugs [32]. According to a report from the Korea Institute for Health and Social Affairs, nutritional imbalances are observed among female adolescents due to body image perception and the consumption of convenience foods. Additionally, young women face a variety of challenges in managing their diet, while adult women in Korea also experience nutritional imbalances [33]. According to the KDRI application report, it has been suggested that an increased prevalence of the population with lower folate intake than the estimated average requirement (EAR) is observed in Korean women aged 19–29 years who live in single-person households, have a higher rate of eating out, and have a low income [29]. However, the data from KNHANES 2016–2018 did not include serum homocysteine levels using fresh specimens [34,35]. A previous study conducted in Korea revealed that these assays are underutilized in the country [10]. Another previous study performed on Korean women to assess folate deficiency had its limitations, such as the use of only serum folate assays not traceable to NIBSC 03/178, or the use of other metabolic indicators without serum folate [6,10,18,22,23,35]. Moreover, these studies involved limited numbers of Korean participants or were conducted prior to the COVID-19 pandemic.

Therefore, the aim of this study was to investigate folate deficiency in Korean women by examining both serum folate and serum homocysteine levels, which serve as a metabolic indicator for folate deficiency during the recent period of the COVID-19 pandemic [8]. Furthermore, we aimed to determine the cut-off for homocysteine levels to predict folate deficiency. This information could provide fundamental knowledge for identifying women at risk of folate deficiency, thereby enabling the implementation of nutritional support programs to improve maternal and child health as part of public health initiatives [22].

## 2. Materials and Methods

### 2.1. Study Subjects

We conducted a retrospective review of laboratory data for serum folate and homocysteine test results obtained from the laboratory information system of Green Cross Laboratories. The data review spanned from 1 January 2020 to 31 December 2022. Green Cross Laboratories serves as a referral laboratory, providing specimen analysis for serum folate and homocysteine tests upon request from local clinics and hospitals in Korea. Test results that solely included homocysteine were excluded from the analysis. Given that the objective of this study was to investigate the prevalence of folate deficiency, repeated test results from the same individual were excluded, with the exception of the initial one. To ensure privacy and confidentiality, all data were anonymized before conducting any statistical analysis.

### 2.2. Definitions

Folate deficiency was determined using the cut-off values recommended by the WHO guidelines [7,8]. For serum folate, the cut-off values for folate deficiency were set at <4 ng/mL (<10 nmol/L) based on the WHO criteria, which consider homocysteine concentrations as a metabolic indicator [7]. Additionally, a cut-off of <3 ng/mL (<6.8 nmol/L) for serum folate was applied, indicating values at which megaloblastic anemia is more likely to occur according to the WHO criteria [7,8]. For homocysteine levels, suspicion of folate deficiency was defined when the serum homocysteine level was ≥15.4 µmol/L.

The study focused on women of reproductive age, categorized into the following age groups: 20–24, 25–29, 30–34, 35–39, 40–44, and 45–49 years. Geographical regions were classified into six groups based on administrative districts and their proximity [36]. The groups were: (1) Seoul, Incheon, and Gyeonggi-do, (2) Gangwon-do, (3) Chungcheong province, (4) Jeolla province, (5) Gyeongsang province, and (6) Jeju Island [36].

To investigate the utilization of serum homocysteine assay in women of reproductive age for assessing folate deficiency and examining differences in folate status and associated factors, the patients were divided into two groups. Group 1 consisted of patients who underwent testing solely for serum folate, while Group 2 included patients who underwent a simultaneous measurement of serum homocysteine.

The number of patients managed with folate deficiency anemia in Korea was determined by analyzing the relevant International Statistical Classification of Diseases and Related Health Problems (ICD-10-CM) codes (D52) obtained from the Healthcare Big-data Hub by the Health Insurance Review and Assessment Service (HIRA) [37]. Unfortunately, the database did not provide information on the number of tests specifically for serum folate assays, as HIRA grouped different types of vitamins under a single test code for reimbursement purposes.

### 2.3. Analytical Methods

Serum homocysteine levels were measured using Architect Homocysteine assay kits (Abbott Laboratories, Chicago, IL, USA) on Architect i2000SR analyzers (Abbott Laboratories, Singapore), which were traceable to an internal reference standard of S-adenosyl-L-homocysteine in phosphate buffer. Serum folate analysis was conducted using electrochemiluminescence immunoassay with Elecsys Folate assay kits (Roche, Mannheim, Germany) on Cobas 8000 e801 analyzers (Roche, Mannheim, Germany), which were traceable to NIBSC 03/178. The analytical measurement range (AMR) for the serum homocysteine assay was 1.0 to 50.0 µmol/L, while the AMR for the serum folate assay was 0.6 to 20.0 ng/mL. For serum folate, values above the measuring range were reported as >40.0 ng/mL for 2-fold diluted samples, following the manufacturer’s instructions. The reference interval for serum homocysteine, suggested by the manufacturer and verified using clinical specimens from the laboratory, was <15.4 µmol/L [38]. To ensure accuracy, the assays participated in quality assurance programs and underwent proficiency tests provided by the Korean Association of External Quality Assessment Service and the College of American Pathologists. No changes were made to the analytical methods during the study period.

### 2.4. Statistical Analysis

We conducted a comprehensive investigation to determine the prevalence of folate deficiency and increased homocysteine levels in relation to various factors, including age group, tested year, geographical regions, and groups with or without simultaneous measurement of serum homocysteine (Group 1 and Group 2) during the study period. Analysis of variance (ANOVA) tests were employed to assess the differences in serum folate levels among different age groups, tested years, geographical regions, and groups with or without simultaneous measurement of serum homocysteine. Chi-squared tests were used to compare the prevalence of folate deficiency among age groups, tested years, geographical regions, and groups with or without simultaneous measurement of serum homocysteine. Furthermore, multiple logistic regression analysis was conducted to investigate the factors associated with folate deficiency. Receiver operating characteristic (ROC) curve analysis was performed to determine the optimal cut-off for homocysteine levels in defining folate deficiency. Two-by-two contingency tables were analyzed to estimate the agreement between serum homocysteine and serum folate in identifying folate deficiency [39]. Statistical significance was considered at a *p*-value < 0.05, and MedCalc Statistical Software version 20.216 (MedCalc Software Ltd., Ostend, Belgium) and SAS version 9.4 (SAS Institute, Inc., Cary, NC, USA) were utilized for the statistical analyses. Additionally, R software (version 4.2.2; http://www.R-project.org/; accessed on 7 June 2023) was employed to generate maps illustrating the number of patients tested for serum folate and the prevalence of folate deficiency across Korea, as well as forest plots presenting odds ratios for factors associated with folate deficiency.

## 3. Results

### 3.1. Baseline Characteristics of the Subjects

**Figure 1:**
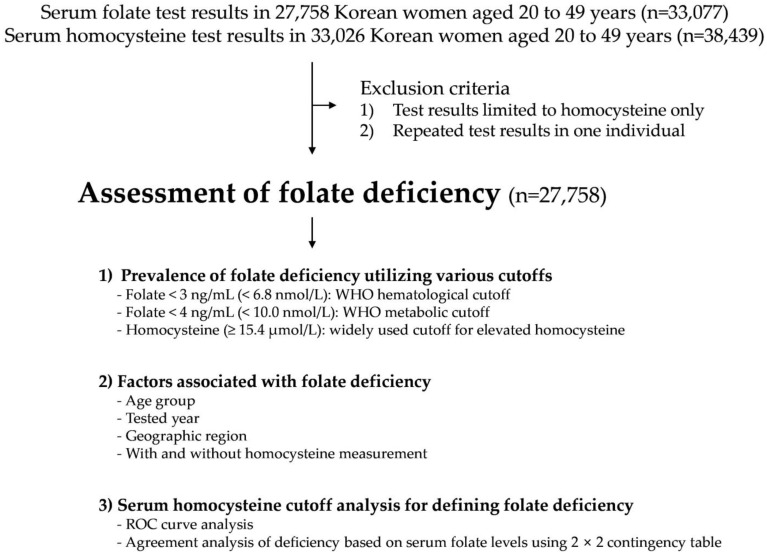
Study scheme.

During the three-year study period, we collected data from 33,077 serum folate and 38,439 serum homocysteine test results conducted on Korean women aged 20 to 49 years. After excluding certain cases, a total of 25,429 women without simultaneous measurement of serum homocysteine (Group 1, 91.6%) and 2329 women with simultaneous measurement of serum folate and homocysteine levels (Group 2, 8.4%) were included in the analysis. The study scheme and baseline characteristics are summarized in Figure 1 and Table 1, respectively.

Among the study subjects, the number of women tested for serum folate varied by age group. Women aged 20 to 24 years accounted for 8.1% (the lowest proportion), while women aged 45 to 49 years constituted 27.7% (the highest proportion) of the study population. Over the three-year study period, there was a notable 35% increase in the number of patients undergoing serum folate assays in 2021 compared to 2020, while the number of patients remained similar in 2022 compared to 2021. In terms of geographical regions, the majority of the patients were tested in Seoul, Incheon, and Gyeonggi-do (68.2%), followed by Gyeongsang province (20.7%). However, only 125 women were tested for serum folate on Jeju Island.

### 3.2. Prevalence of Folate Deficiency and Associated Factors

The overall prevalence of folate deficiency was 5.4% for the cut-off of serum folate <3.0 ng/mL and 12.5% for the cut-off of serum folate <4.0 ng/mL. The levels of serum folate and the prevalence of folate deficiency according to each cut-off for serum folate and homocysteine levels are summarized in Table 2. Serum folate levels showed significant differences among the age groups, tested years, geographical regions, and groups with and without simultaneous measurement of serum homocysteine (all *p* < 0.0001).

**Figure 2:**
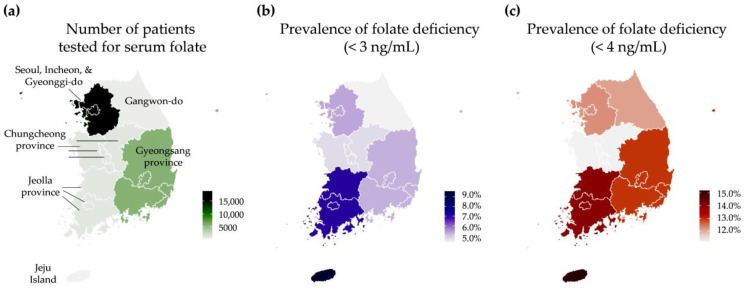
Folate deficiency in Korea. (**a**) Number of patients tested for serum folate. (**b**) Prevalence (%) of folate deficiency (<3 ng/mL) among the tested patients. (**c**) Prevalence (%) of folate deficiency (<4 ng/mL) among the tested patients.

The prevalence of folate deficiency, defined as serum folate levels <3 ng/mL and <4 ng/mL, varied significantly across the age groups, tested years, and groups with and without simultaneous measurement of serum homocysteine (all *p* < 0.0001). The prevalence of folate deficiency differed significantly among the geographical regions when the cut-off was <3 ng/mL (*p* < 0.05) but not when the cut-off was <4 ng/mL (*p* ≥ 0.05). Notably, the prevalence of folate deficiency was higher in younger age groups compared to older groups, with the highest prevalence observed in the 20 to 24-year-old group (11.2% for <3 ng/mL and 24.9% for <4 ng/mL). Over the study period, the prevalence of folate deficiency slightly decreased from 5.9% in 2020 to 4.5% in 2022 (cut-off <3 ng/mL) and from 13.1% in 2020 to 11.1% in 2022 (cut-off <4 ng/mL). Additionally, the prevalence of folate deficiency was higher in the Group 1 patients without simultaneous measurement of serum homocysteine (5.5% for <3 ng/mL and 12.8% for <4 ng/mL) compared to the Group 2 patients with simultaneous measurement of serum homocysteine (3.6% for <3 ng/mL and 9.1% for <4 ng/mL, *p* <0.0001). The number of tested patients and the prevalence of folate deficiency by geographical region in Korea are presented as maps in Figure 2.

**Figure 3:**
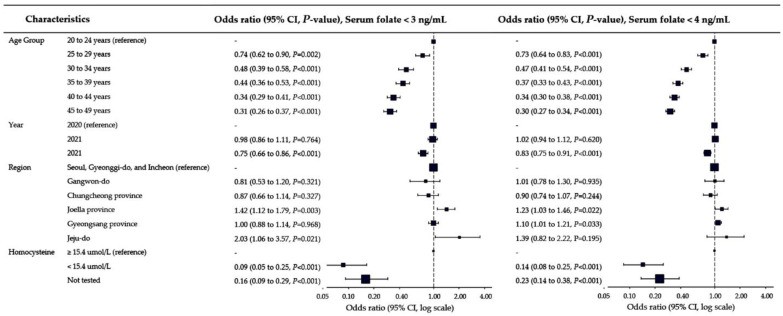
Multiple logistic regression analysis for factors associated with folate deficiency.

Multiple logistic regression analysis was conducted to explore factors associated with folate deficiency. Age group, tested year, geographical region, and test groups (with/without homocysteine) were found to be significantly associated with folate deficiency (Figure 3, all *p* < 0.05). Women in the younger age groups had a higher likelihood of experiencing folate deficiency. Women in age groups older than 20 to 24 years, those tested in 2022 compared to 2020, and individuals with homocysteine levels <15.4 µmol/L compared to ≥15.4 µmol/L were less likely to have folate deficiency. Among the different geographical regions, women tested in Jeolla province, Gyeongsang province, and on Jeju Island were more likely to have folate deficiency than those tested in the Seoul, Incheon, and Gyeonggi-do area.

### 3.3. Number of Patients Managed with Folate Deficiency in the Public Database

**Figure 4:**
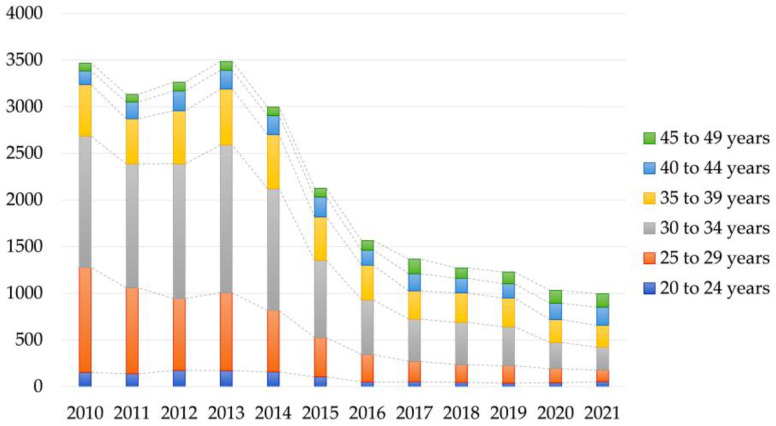
Number of women managed for folate deficiency anemia (D52) in Korea from 2010 to 2021 (data from the HIRA Bigdata Open Portal of the Health Insurance Review and Assessment Service) [38].

Although the number of patients undergoing serum folate assays was not available in the database provided by HIRA, we were able to obtain data on the number of patients managed for folate deficiency anemia (D52) over the past 10 years (Figure 4). The data showed a gradual decrease in the number of women aged 20 to 49 years with folate deficiency anemia, from 3485 patients in 2013 to 996 patients in 2021. Among these patients, the majority were in the age group of 30 to 34 years (39.8%), followed by the age group of 25 to 29 years (22.7%), and the age group of 35 to 39 years (19.4%).

### 3.4. Homocysteine to Define Folate Deficiency

**Figure 5:**
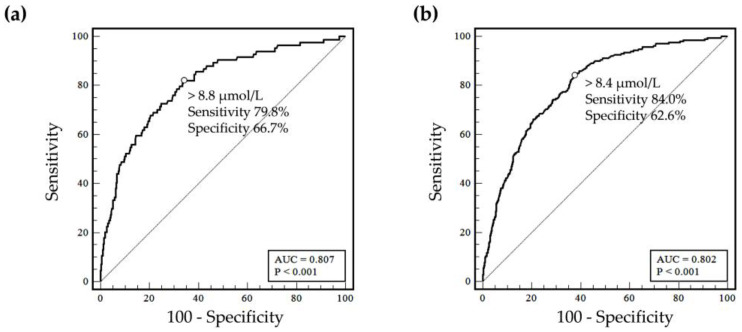
Area under the curve (AUC) of the receiver operating characteristic curve analysis to identify folate deficiency using serum homocysteine. (**a**) ROC analysis to determine the optimal homocysteine level for defining folate deficiency with a cut-off of serum folate <3 ng/mL. (**b**) ROC analysis to determine the optimal homocysteine level for defining folate deficiency with a cut-off of serum folate <4 ng/mL.

The overall prevalence of folate deficiency, determined by the criterion of a serum homocysteine level ≥15.4 µmol/L regardless of the serum folate level, was found to be 2.7% among all women included in the study. To identify the optimal homocysteine level for defining folate deficiency, we conducted a receiver operating characteristic (ROC) analysis (Figure 5). The ROC curve analysis yielded an area under the curve (AUC) of 0.802 (95% confidence interval: 0.772 to 0.831) for a homocysteine level >8.4 µmol/L as a predictor of folate deficiency (defined as <4 ng/mL according to the WHO guidelines for the metabolic indicator of folate deficiency).

The agreement between serum folate and serum homocysteine in the Group 2 patients to identify folate deficiency was assessed using a 2 × 2 contingency table at different cut-offs for the serum biomarkers, as presented in Table 3.

When a homocysteine level ≥15.4 µmol/L was used to define folate deficiency, the positive agreement was 26.6% (95% confidence interval: 17.3 to 38.5). However, when the cut-off values derived from the ROC analyses were applied, the positive agreement increased, while the overall agreement and negative agreement decreased.

## 4. Discussion

We conducted a study to assess the utilization of serum folate and homocysteine as-says among a large population of Korean women of reproductive age who sought medical care at local clinics and hospitals during the recent COVID-19 pandemic. Additionally, we aimed to determine the optimal cut-off for detecting increased homocysteine levels according to the WHO criteria for folate deficiency (serum folate < 4 ng/mL using an analytical method traceable to NIBSC 03/178). Our findings revealed that women in younger age groups, those tested in 2020, and those tested in Jeolla province, Gyeongsang province, and Jeju Island regions had a higher risk of folate deficiency.

In this study, the overall prevalence of folate deficiency and the prevalence by age group were found to be comparable to a previous study conducted in the same laboratory from 2017 to 2019 [22]. The prevalence of folate deficiency in 1747 Korean reproductive women aged 20 to 49 years, as assessed in the KNHANES phase VII (2016–2018) using fresh specimens and based solely on serum folate, was reported as 2.6% for the cut-off < 3.0 ng/mL and 9.8% for <4 ng/mL, which was slightly lower than the prevalence observed in our present study [40]. The analytical method employed to measure serum folate in the KNHANES Phase VII (2016–2018) utilized a chemiluminescent microparticle immunoassay with Architect folate assay kits on Architect i4000SR, which differed from the analytical method employed in our study, but both methods were traceable to NIBSC 03/178. Despite the introduction of the WHO International Standard NIBSC 03/178 and efforts by the International Federation of Clinical Chemistry and Laboratory Medicine (IFCC) and the National Institute of Standards and Technology (NIST) for assay harmonization and standardization, there still exists variability in the results between different measurement methods [19,20]. Therefore, caution should be exercised when interpreting folate deficiency using a common threshold for detecting folate deficiency given the current status of assay standardization and harmonization [20].

During the COVID-19 pandemic period from 2020 to 2022, the prevalence of folate deficiency did not show significant fluctuations, but a substantial proportion of patients still experienced folate deficiency. Our study found that the highest prevalence was observed in the youngest age group of women, specifically those aged 20 to 24 years, with rates of 11.2% for <3 ng/mL and 24.9% for <4 ng/mL. This pattern was consistent with previous studies conducted in the same laboratory and the KNHANES phase VII (2016–2018), which also reported a higher prevalence of folate deficiency in the youngest age group (5.5% for <3 ng/mL and 19.7% for <4 ng/mL) [22,40,41]. These findings highlight the increased risk of folate deficiency in younger women, emphasizing the need for targeted public health plans and nutritional supplements for maternal and child health, particularly focusing on younger women [18]. In 2006, the WHO and the United Nations (UN) Food and Agricultural Organization published guidelines to assist countries in implementing fortification strategies based on national food consumption patterns and globally recommended nutrient forms [1]. Data from NHANES in the USA, a country that has implemented a folic acid fortification program for over 20 years but has the same recommended nutrient intake as Korea, reported an overall prevalence of folate deficiency using a cut-off of serum folate <7 nmol/L (<3.08 ng/mL, using a conversion factor of 1 ng/mL = 2.265 nmol/L) [42]. The NHANES study employed different measurement methods, specifically a radioassay and liquid chromatography-tandem mass spectrometry, which were different from the analytical methods used in the current study as well as those utilized in KNHANES [40,42]. According to the PRiDE study, a recent prospective cohort study conducted in the UK between 2012 and 2018, in a country with a folic acid fortification program, the prevalence of folate deficiency, defined as a serum folate cut-off of <10 nmol/L (<4 ng/mL), was 1.3% among pregnant women aged 18 to 45 years within the first 16 weeks of gestation [43]. Although women in Korea receive medical and financial support from the government, including folate supplementation, such support is typically provided after pregnancy has been confirmed by an obstetrician [41]. Although the exact information on the current intake of folate supplementation is unknown, supplementation of folate 400 μg/day has been recommended for women of reproductive age since 2010 in Korea [23,28,29]. This is the same recommendation given by the International Federation of Gynecology and Obstetrics [44]. Considering the common occurrence of folate deficiency in younger women and the importance of folate status during early pregnancy for fetal neural development, it is crucial to implement public health plans that increase awareness about the significance of folate supplementation, with a focus on educating and targeting younger women before they become pregnant [1,41]. Future studies need to incorporate data on natural, daily diets and dietary supplements, as well as folate biomarkers, in large Korean populations.

The population included in our study was older compared to the population man-aged with folate deficiency anemia (D52) in the HIRA database. Among the patients tested for serum folate assays in our study, the largest group consisted of women in their 40s, while the highest prevalence of folate deficiency was observed in women in their 20s. On the other hand, the largest group of patients managed with folate deficiency anemia (D52) in the HIRA database was women aged 25 to 35 years, which aligns with the age range typically associated with childbirth in Korea [45]. These differences in patient populations may be attributed to various factors, such as the healthcare-related behaviors of patients and the clinician’s preference for using the ICD-10-CM code for managing patients with reimbursable folate deficiency anemia [46,47]. For instance, in our study population of women in their 40s, they may have undergone folate testing for different medical conditions when visiting local clinics and hospitals. On the other hand, Korean clinicians might use the reimbursable code for D52 folate deficiency anemia in their daily practice to manage women who come for prenatal visits, while physicians might not utilize the D52 code for folate deficiency anemia when treating other patients for various medical conditions. However, detailed clinical information was limited both in our study population and the public database. Further research is needed to investigate folate deficiency anemia using comprehensive clinical information.

In the current study, the utilization of homocysteine assays for managing women of reproductive age in Korea was found to be low, consistent with a previous study conducted in Korea [10]. The prevalence of folate deficiency in Group 1 patients with serum folate only was higher compared to Group 2, contradicting previous knowledge that homocysteine is a sensitive biomarker for folate deficiency [6]. The underutilization of the homocysteine assay may have introduced bias in the study results. The proportion of patients with elevated homocysteine levels but without a decrease in serum folate might be influenced by recent dietary intake or other comorbidities [6,7]. In this study, we investigated the optimal level of serum homocysteine to define folate deficiency. The cut-off value suggested in this study (>8.4 µmol/L for a metabolic indicator of folate deficiency of <4 ng/mL) was lower than the manufacturer’s reference interval (≥15.4 µmol/L), which is a traditionally widely used cut-off. Considering the results of the AUC and agreement, the limited assay standardization, underutilization observed in this study, and the lack of information on other clinical factors and laboratory findings that may influence both biomarkers, further studies are needed to determine the optimal cut-off for homocysteine levels in identifying folate deficiency in diverse populations using different analytical methods.

One limitation of the present study was the lack of clinical information associated with folate deficiency, such as hematologic parameters, vitamin B12 status, comorbidities, medication, and supplement use [6]. However, the assessment of folate deficiency in this study was conducted according to the WHO guidelines, using serum folate and homocysteine as biomarkers. To mitigate selection bias, we compared the prevalence of folate deficiency in our study population with information from public databases in Korea. The number of patients tested for serum folate assays and identified as having folate deficiency in our study population was consistent with the number of women managed for folate deficiency in the public database across Korea. These findings can be generalized to populations of women of reproductive age visiting local clinics and hospitals. Future studies with comprehensive clinical information are needed to further investigate the utilization of biomarkers for serum folate status and their association with maternal and child health outcomes.

A strength of this study is the large sample size, particularly during the recent COVID-19 pandemic period. The results of this study can serve as a foundation for developing guidelines for nutritional support in public health. Our findings highlight the importance of analyzing laboratory data to assess the utilization and prevalence of nutritional deficiencies, providing valuable information for public health initiatives [46,47].

## 5. Conclusions

This study investigated the prevalence of folate deficiency by assessing the utilization of serum folate and homocysteine assays in a large population of Korean women of reproductive age during the COVID-19 pandemic period. The prevalence of folate deficiency was determined based on the current criteria set by the WHO, and it was found to be common among younger women. Future studies should further investigate the optimal serum homocysteine levels for defining folate deficiency, taking into account the standardization and harmonization of analytical methods, as well as comprehensive clinical information. The findings of this study provide recent information on folate deficiency in the Korean population, which can guide the development of appropriate nutritional strategies for public health.

## Figures and Tables

**Table 1:** Baseline characteristics of the 27,758 study subjects.

Characteristics		Data
Age	Age, years (median, IQR)	40.6 (33.1 to 45.6)
	20 to 24 years (n, %)	2242 (8.1%)
	25 to 29 years (n, %)	2755 (9.9%)
	30 to 34 years (n, %)	3252 (11.7%)
	35 to 39 years (n, %)	4764 (17.2%)
	40 to 44 years (n, %)	7044 (25.4%)
	45 to 49 years (n, %)	7701 (27.7%)
Tested year	Tested in 2020 (n, %)	7573 (27.3%)
	Tested in 2021 (n, %)	10,261 (37.0%)
	Tested in 2022 (n, %)	9924 (35.8%)
Geographical region	Seoul, Incheon, and Gyeonggi-do (n, %)	18,938 (68.2%)
	Gangwon-do (n, %)	577 (2.1%)
	Chungcheong province (n, %)	1231 (4.4%)
	Jeolla province (n, %)	1147 (4.1%)
	Gyeongsang province (n, %)	5740 (20.7%)
	Jeju Island (n, %)	125 (0.5%)
Group	Group 1 (serum folate only; n, %)	25,429 (91.6%)
	Group 2 (serum folate and homocysteine; n, %)	2329 (8.4%)
Serum biomarker	Serum folate, ng/mL (median, IQR)	8.10 (5.30 to 13.10)
	Serum homocysteine, µmol/L (median, IQR)	7.94 (6.65 to 9.49)

Abbreviations: IQR, interquartile range.

**Table 2:** Serum folate levels by age, tested year, geographical regions, and test groups with and without simultaneously measured homocysteine.

Characteristics		Serum Folate (ng/mL)			Folate Deficiency (<3 ng/mL)			Folate Deficiency (<4 ng/mL)		
		Mean	SD	*p*-Value	n	%	*p*-Value	n	%	*p*-Value
Age group	20 to 24 years	7.40	5.58	<0.0001	251	11.2	<0.0001	558	24.9	<0.0001
	25 to 29 years	8.71	6.69		233	8.5		531	19.3	
	30 to 34 years	10.81	8.25		179	5.5		429	13.2	
	35 to 39 years	11.60	8.50		248	5.2		525	11.0	
	40 to 44 years	11.02	8.10		290	4.1		712	10.1	
	45 to 49 years	10.95	7.53		293	3.8		707	9.2	
Tested year	Tested in 2020	9.90	7.20	<0.0001	448	5.9	<0.0001	991	13.1	<0.0001
	Tested in 2021	10.38	7.77		599	5.8		1373	13.4	
	Tested in 2022	11.07	8.25		447	4.5		1098	11.1	
Geographical region	Seoul, Incheon, and Gyeonggi-do	10.78	8.05	<0.0001	1016	5.4	0.0159	2328	12.3	0.2003
	Gangwon-do	10.12	7.76		25	4.3		70	12.1	
	Chungcheong province	9.64	6.62		58	4.7		138	11.2	
	Jeolla province	9.62	6.76		82	7.1		161	14.0	
	Gyeongsang province	9.96	7.38		301	5.2		746	13.0	
	Jeju island	10.28	7.66		12	9.6		19	15.2	
Group	Group 1 (serum folate only)	10.36	7.73	<0.0001	1410	5.5	<0.0001	3249	12.8	<0.0001
	Group 2 (serum folate and homocysteine)	11.98	8.55		84	3.6		213	9.1	

Abbreviations: SD, standard deviation. The *p*-values for serum folate levels by characteristics were from the analysis using ANOVA and t-test, and folate deficiency by characteristics was from the analysis using the chi-squared test.

**Table 3:** Agreement of folate deficiency between the serum folate cut-off and the homocysteine cut-off.

Homocysteine Cut-off	Folate Deficiency	Folate < 3 ng/mL		Folate < 4 ng/mL	
		Deficiency (n)	No Deficiency (n)	Deficiency (n)	No Deficiency (n)
Transferred from the manufacturer’s information, ≥15.4 µmol/L	Deficiency (n)	17	47	25	39
	No deficiency (n)	67	2198	188	2077
	Overall agreement (%, 95% CI)	95.1 (94.2 to 95.9)		90.3 (89.0 to 91.4)	
	Positive agreement (%, 95% CI)	26.6 (17.3 to 38.5)		39.1 (28.1 to 51.3)	
	Negative agreement (%, 96% CI)	97.0 (96.3 to 97.7)		91.7 (90.5 to 92.8)	
ROC analysis, >8.4 µmol/L	Deficiency (n)	72	898	179	791
	No deficiency (n)	12	1347	34	1325
	Overall agreement (%, 95% CI)	60.9 (58.9 to 92.9)		64.6 (62.6 to 66.5)	
	Positive agreement (%, 95% CI)	85.7 (79.7 to 91.6)		84.0 (78.5 to 88.3)	
	Negative agreement (%, 96% CI)	60.0 (58.0 to 62.0)		62.6 (60.5 to 64.7)	
ROC analysis, > 8.8 µmol/L	Deficiency (n)	67	748	160	655
	No deficiency (n)	17	1497	53	1461
	Overall agreement (%, 95% CI)	79.8 (70.0 to 87.0)		75.1 (68.9 to 80.4)	
	Positive agreement (%, 95% CI)	66.7 (64.7 to 68.6)		69.0 (67.0 to 71.0)	
	Negative agreement (%, 96% CI)	67.2 (65.2 to 69.0)		69.6 (67.7 to 71.4)	

Abbreviations: CI, confidence interval; ROC, receiver operating characteristic curve.

## Data Availability

The datasets generated and analyzed during the current study are available from the corresponding authors upon reasonable request.
